# When Morphology Falls Short: ATP1B1::PRKACB Fusion in a Morphologically Atypical Pancreatic Intraductal Oncocytic Neoplasm

**DOI:** 10.7759/cureus.117098

**Published:** 2026-09-28

**Authors:** Suyeon Han, Ying-Hsia Chu, Robben Schat, Ismail Elbaz Younes, David Brauer, David Cartwright, Amin Khalid, Kevin Turner, Byoung Uk Park

**Affiliations:** 1 Department of Pathology, Jeonbuk National University Medical School, Jeonju, KOR; 2 Department of Laboratory Medicine and Pathology, University of Minnesota, Minneapolis, USA; 3 Department of Radiology, University of Minnesota, Minneapolis, USA; 4 Department of Surgery, University of Minnesota, Minneapolis, USA

**Keywords:** atp1b1::prkacb fusion, intraductal oncocytic papillary neoplasm, iopn, next-generation sequencing, pancreatic intraductal neoplasm

## Abstract

Intraductal oncocytic papillary neoplasm (IOPN) is a rare pancreatic intraductal neoplasm characterized by distinctive oncocytic morphology and recurrent gene fusions involving PRKACA or PRKACB. While conventional IOPN exhibits complex arborizing papillae lined by oncocytic epithelium, morphologically atypical lesions may lack these hallmark features and demonstrate overlap with intraductal papillary mucinous neoplasm (IPMN).

A 76-year-old man presented with a pancreatic cystic lesion that remained radiographically stable over five years of surveillance and was initially considered indeterminate, though it was favored to represent a side-branch IPMN. Persistent main pancreatic duct dilation to 10 mm and a cyst measuring approximately 4.5 cm prompted multidisciplinary evaluation and subsequent pancreaticoduodenectomy. Histologic examination revealed a 3.4-cm intraductal neoplasm predominantly composed of foveolar-type and pancreatobiliary-type epithelium, with focal oncocytic differentiation and extensive high-grade dysplasia. The classic complex arborizing architecture characteristic of conventional IOPN was absent, and no invasive carcinoma was identified. RNA-based next-generation sequencing identified an ATP1B1::PRKACB fusion, while KRAS and GNAS alterations were not detected, strongly supporting its classification as a morphologically atypical intraductal oncocytic neoplasm within the IOPN spectrum.

This case underscores the diagnostic value of integrating morphologic and molecular assessment when oncocytic differentiation is present, but conventional IOPN morphology is incompletely developed. Additionally, it provides uncommon longitudinal documentation of an IOPN-spectrum lesion that remained radiographically stable for five years.

## Introduction

Intraductal oncocytic papillary neoplasm (IOPN) is a rare, mass-forming intraductal neoplasm of the pancreas that accounts for approximately 3-5% of pancreatic intraductal neoplasms [1]. Adsay et al. first delineated the entity in 1996, reporting 11 cases [2]. In the 2010 World Health Organization (WHO) classification, these tumors were initially categorized as an oncocytic subtype of intraductal papillary mucinous neoplasm (IPMN) because of overlapping features [3]. As additional morphologic and molecular evidence accumulated, the 2019 WHO classification recognized IOPN as a distinct entity separate from IPMN and intraductal tubulopapillary neoplasm (ITPN) [4,5].

IOPN predominantly occurs in middle-aged to older adults, with a reported mean age at diagnosis of approximately 58-59 years [3,4]. In a systematic review of 409 patients, men accounted for 59.9% of cases, corresponding to a male-to-female ratio of approximately 1.5:1 [4], although sex distributions have varied across published series [1,3,4]. The pancreatic head is the most commonly involved site [3,4]. Tumors are often relatively large at diagnosis, with a mean size of approximately 4.5 cm, and up to 44% are discovered incidentally [3,4]. Histologically, conventional IOPN is characterized by complex arborizing papillae supported by delicate fibrovascular cores and lined by several cell layers with abundant granular eosinophilic cytoplasm, round, relatively uniform nuclei, and a single prominent nucleolus, often eccentrically located [3,4]. Characteristic intraepithelial lumina may impart a cribriform appearance [1,3]. Mucin production is generally not prominent and is usually limited to occasional luminal pools, intracellular droplets, scattered goblet cells, or intraepithelial luminal spaces [1,4].

The most discriminating evidence separating IOPN from conventional IPMN is its molecular profile. Singhi et al. identified an ATP1B1::PRKACB, DNAJB1::PRKACA, or ATP1B1::PRKACA fusion in all 20 surgically resected pancreatic IOPNs examined and detected corresponding fusions in associated invasive carcinomas and matched preoperative cyst-fluid specimens [6]. The same rearrangements were not identified in a separate cohort of 126 non-IOPN pancreatobiliary lesions, including pancreatic ductal adenocarcinoma, conventional IPMN, mucinous cystic neoplasm, and cystic neuroendocrine tumor [6]. In a systematic review, a fusion involving PRKACA or PRKACB was demonstrated in all 68 cases in which fusion status was assessed [4]. These rearrangements are thought to dysregulate protein kinase A-related signaling, which has been proposed as a mechanism contributing to oncocytic differentiation [1]. In contrast, KRAS and GNAS alterations, which are common in conventional IPMN, are less frequent in conventional IOPN [4,6,7]. However, not all IOPNs demonstrate this fully developed morphologic phenotype. Tanaka et al. described atypical morphology in two principal forms: (1) an oncocytic component lacking characteristic cytoarchitecture, including complex arborizing papillae and prominent nucleoli; and (2) admixture of an oncocytic component with a non-oncocytic IPMN-like component of gastric, intestinal, or pancreatobiliary type [5]. PRKACA/PRKACB fusions remained detectable in many such lesions, although at lower frequencies than in morphologically typical tumors. Using fluorescence in situ hybridization, Tanaka et al. identified PRKACA/PRKACB fusions in 15 of 19 atypical IOPNs (79%), compared with three of three morphologically typical tumors (100%) [5]. Itoh et al., using reverse-transcription polymerase chain reaction, detected fusions in 32 of 32 typical IOPNs (100%), 13 of 28 atypical oncocytic neoplasms (46%), and none of 20 non-oncocytic controls, a statistically significant difference (P < 0.001) [7]. In some morphologically mixed lesions, Itoh et al. found that the fusion was restricted to the oncocytic component. At the same time, IPMN-associated driver alterations were present throughout the lesion, leading the authors to propose that acquisition of the fusion may, in some cases, promote subclonal evolution toward an oncocytic phenotype within an IPMN [7].

We report a radiographically stable pancreatic intraductal lesion with morphologic features overlapping those of IOPN and IPMN and focal oncocytic differentiation, for which molecular testing strongly supported classification within the IOPN spectrum. The case illustrates the diagnostic gray zone between these entities and highlights the value of integrated morphologic and molecular assessment when histology alone cannot confidently classify a pancreatic intraductal neoplasm.

## Case presentation

A 76-year-old man with a five-year history of a pancreatic cystic lesion had been followed with annual surveillance imaging. The lesion was considered indeterminate but was favored radiographically to represent a side-branch IPMN. Germline testing had identified a heterozygous SPINK1 c.101A>G (p.Asn34Ser; N34S) variant; however, the patient had no personal or family history of pancreatitis. Serial magnetic resonance imaging (MRI), including the most recent surveillance study, demonstrated a stable 2.3 × 4.5 × 4.2 cm multiloculated cystic lesion in the pancreatic head, without a solid component or enhancing mural nodule, and a persistently dilated main pancreatic duct measuring up to 10 mm (Figure 1).

**Figure 1:**
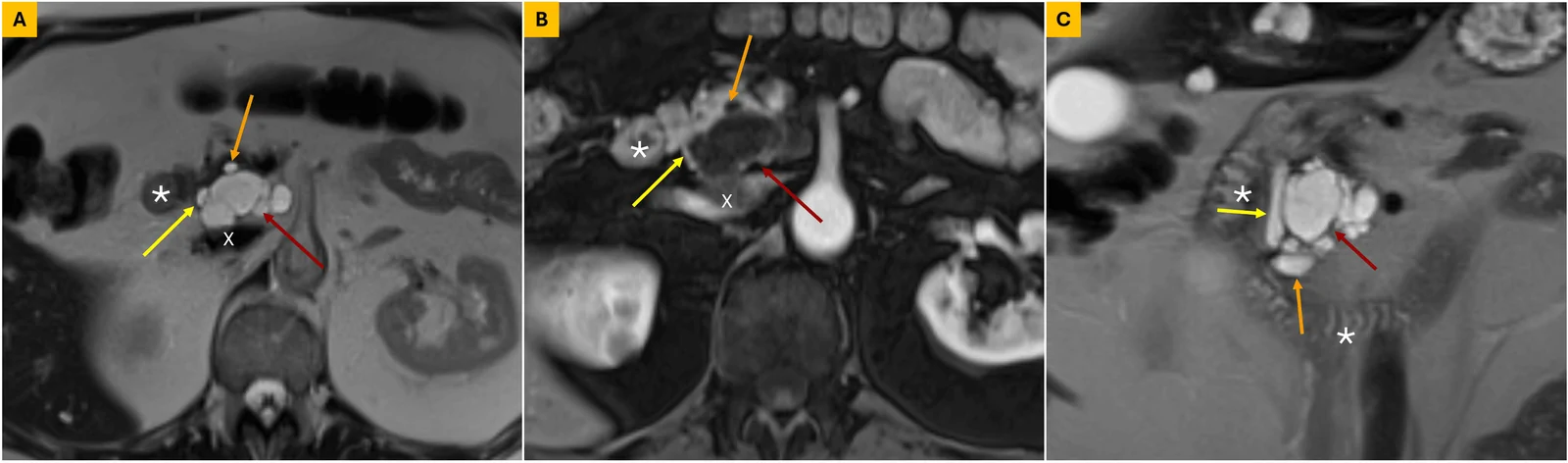
Magnetic resonance imaging appearance of the pancreatic cystic lesion. Axial non-fat-saturated T2-weighted (A), axial late arterial-phase contrast-enhanced T1-weighted (B), and coronal non-fat-saturated T2-weighted (C) images demonstrate a 2.3 × 4.5 × 4.2 cm multilobulated, multilocular cystic lesion with thin internal septa centered in the pancreatic head (red arrows). No solid component or enhancing mural nodule is identified. The prominent main pancreatic duct (orange arrows) lies anterior to the lesion on the axial images and inferior to it on the coronal image. The common bile duct (yellow arrows) courses along the right lateral aspect of the lesion. Neither duct demonstrates focal obstruction by the lesion. White × symbols indicate the inferior vena cava in A and B. White asterisks indicate the second portion of the duodenum in A and B and the second and third portions of the duodenum in C.

Due to the persistent main pancreatic duct dilation to 10 mm and the size of the cystic lesion, a follow-up triple-phase pancreas-protocol computed tomography (CT) examination was performed approximately three months later. CT again demonstrated a similarly sized, approximately 4.5-cm, lobulated, thin-septated, hypodense lesion centered in the pancreatic head/neck, without a solid component or an enhancing mural nodule, and without significant interval change from the preceding MRI. The lesion remained indeterminate but was again favored to represent a side-branch IPMN. Contrast-enhanced MRI with magnetic resonance cholangiopancreatography (MRCP) was recommended for further characterization (Figure 2).

**Figure 2:**
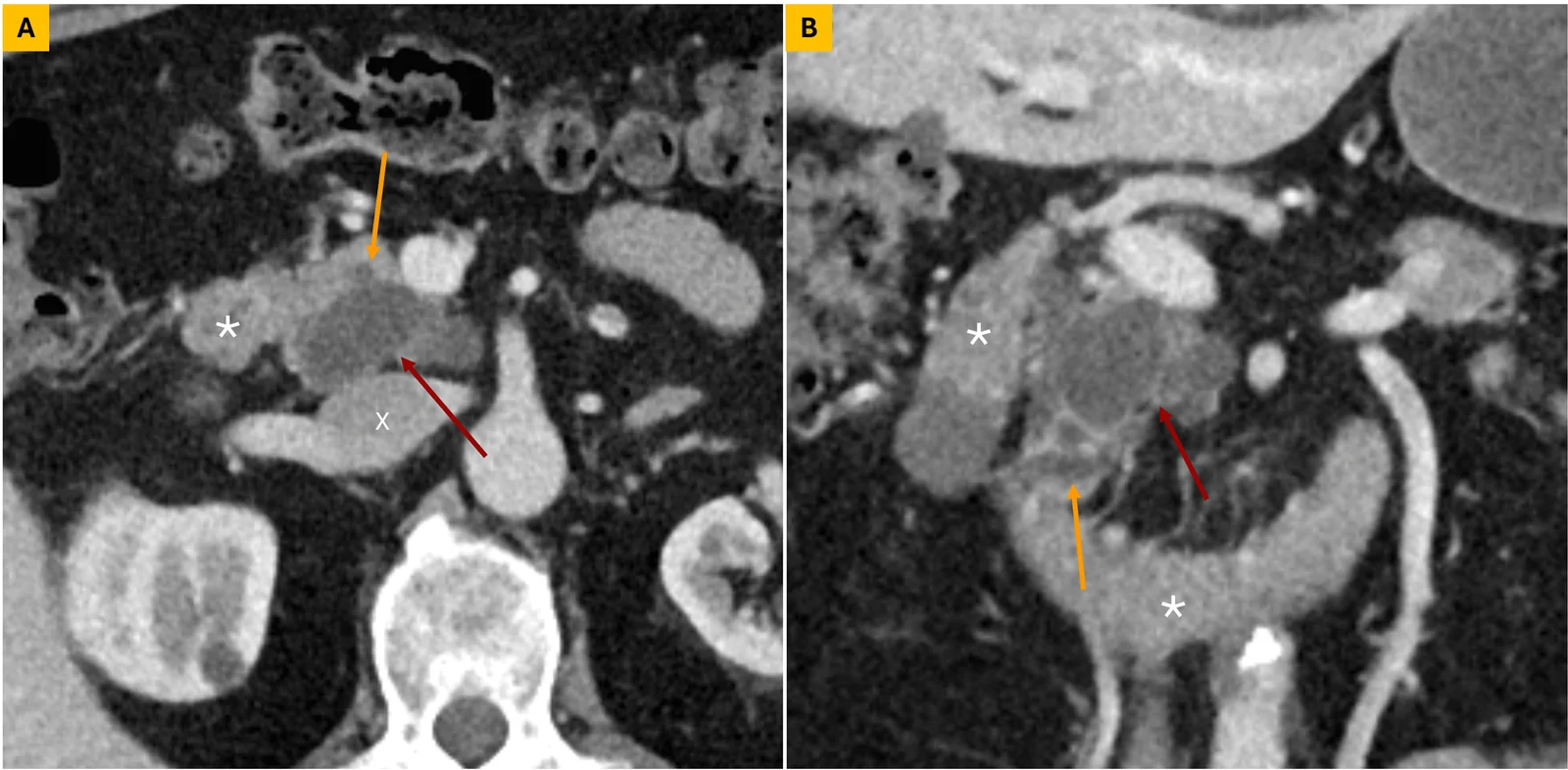
Computed tomography appearance of the pancreatic cystic lesion. Axial (A) and coronal (B) portal venous-phase contrast-enhanced CT images demonstrate a multilobulated, multilocular cystic lesion with thin internal septa centered in the pancreatic head (red arrows). The lesion measures approximately 4.5 cm and contains no solid component or enhancing mural nodule. The prominent main pancreatic duct (orange arrows) courses anterior to the lesion on the axial image and inferior to it on the coronal image, without focal obstruction by the lesion. The white × indicates the inferior vena cava in A; white asterisks indicate the adjacent duodenum in A and B. CT: computed tomography.

Given the persistent main pancreatic duct dilation to 10 mm and the size of the cystic lesion, the case was reviewed at a multidisciplinary tumor board. After reviewing the imaging findings and overall clinical context, surgical resection was recommended. Following a comprehensive discussion of risks, benefits, and alternatives, the patient underwent an open pancreaticoduodenectomy (Whipple procedure) without immediate intraoperative complications. Intraoperative frozen sections of the pancreatic neck and bile duct margins were negative for high-grade dysplasia or malignancy.

Gross examination of the pancreaticoduodenectomy specimen revealed a 3.4 × 2.4 × 2.2 cm predominantly cystic, focally solid lesion arising from and branching off the main pancreatic duct in the pancreatic head (Figure 3). The gross appearance was non-specific and consistent with the preoperative diagnosis of an intraductal cystic neoplasm. The lesion was located less than 0.1 cm from the uncinate margin and vascular groove, 0.1 cm from the posterior soft tissue margin, and 0.4 cm from the pancreatic neck margin. It abutted the bile duct but did not grossly extend into it.

**Figure 3:**
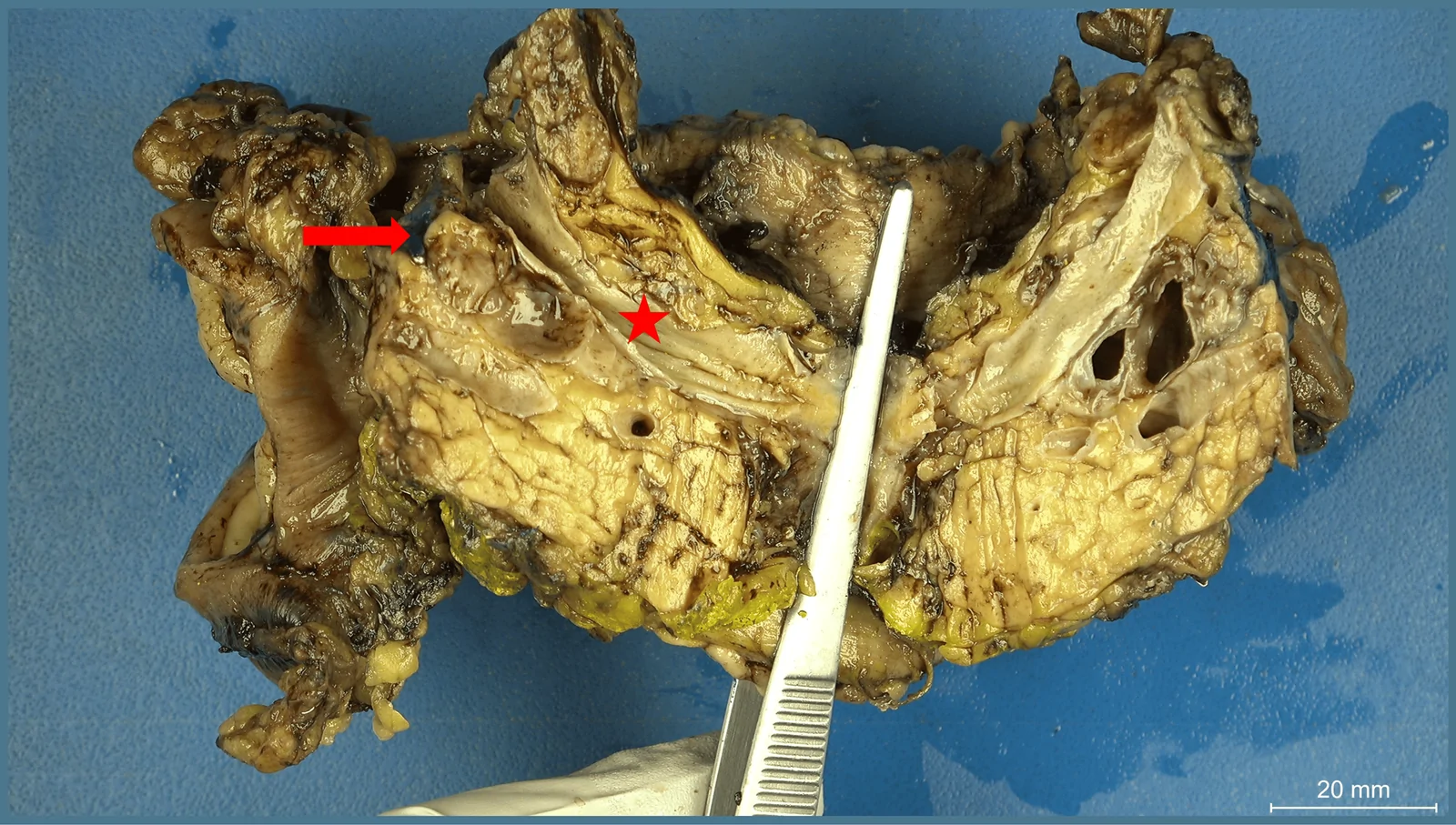
Gross appearance of the pancreatic lesion. The bivalved pancreatic head demonstrates a 3.4 × 2.4 × 2.2 cm predominantly cystic, focally solid lesion (arrow) communicating with and branching from the main pancreatic duct (asterisk). Scale bar = 20 mm.

Histologic examination demonstrated a cystic intraductal neoplasm lined by a combination of foveolar-type and pancreatobiliary-type epithelium, with extensive high-grade dysplasia and focal oncocytic differentiation (Figure 4). The oncocytic component consisted of cells with abundant granular eosinophilic cytoplasm and prominent nucleoli. However, the lesion lacked the complex arborizing papillae and other fully developed architectural features characteristic of conventional IOPN. Morphologically, these findings raised a differential diagnosis between a morphologically atypical intraductal oncocytic neoplasm and an IPMN with focal oncocytic change. No associated invasive carcinoma was identified. All surgical margins were negative for high-grade dysplasia and carcinoma, although the pancreatic neck margin showed incidental low-grade mucinous epithelium. Sixteen regional lymph nodes were negative for carcinoma (0/16).

**Figure 4:**
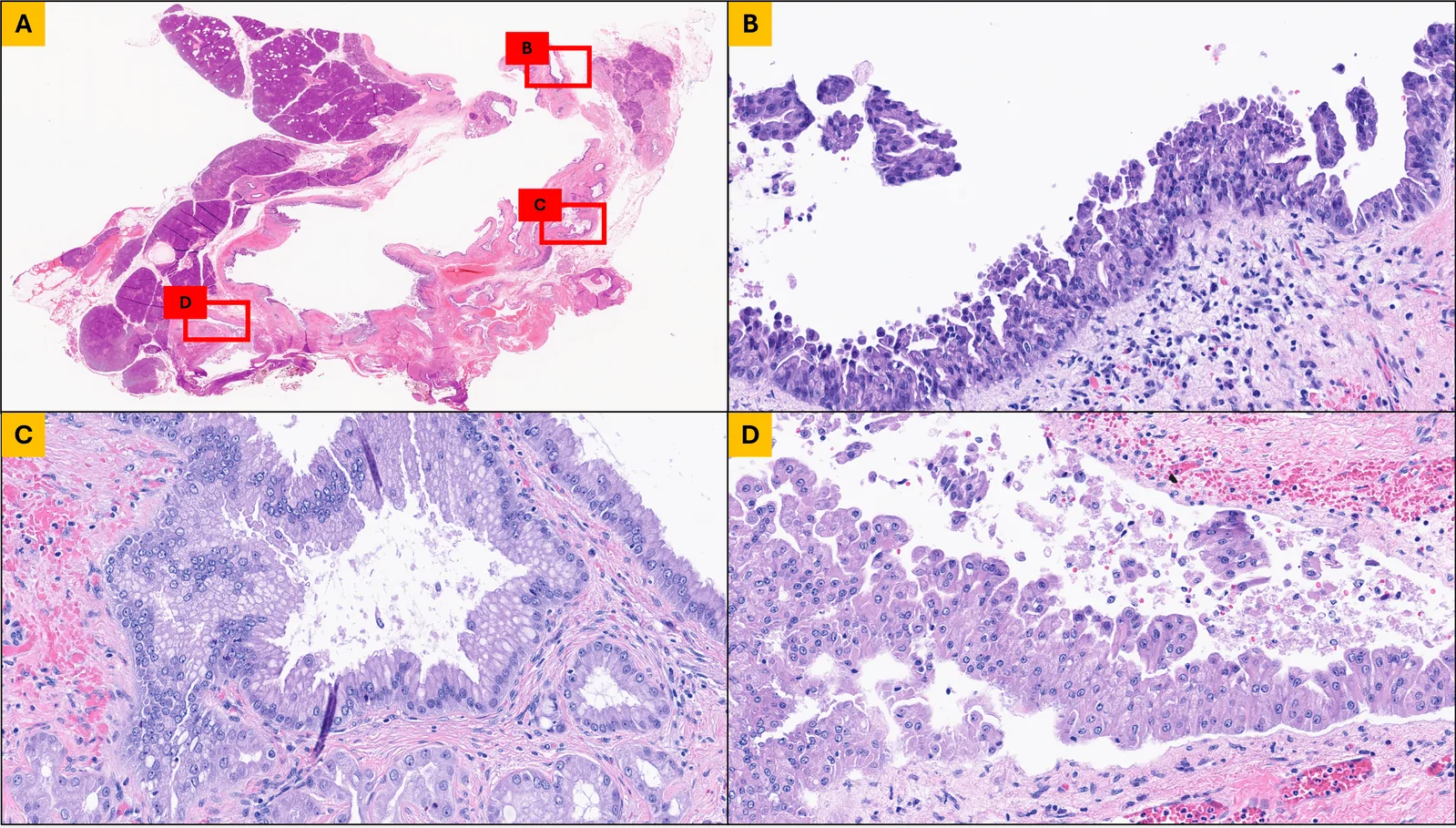
Histologic features of the pancreatic intraductal neoplasm. (A) Low-power overview of the cystic lesion; the boxed regions correspond to the higher-power fields shown in panels B-D. (B) Pancreatobiliary-type epithelium with high-grade dysplasia, characterized by nuclear stratification, loss of polarity, and marked cytologic atypia. (C) Foveolar-type epithelium with abundant pale mucinous cytoplasm. (D) Focus on oncocytic differentiation showing cells with abundant granular eosinophilic cytoplasm and prominent nucleoli. Hematoxylin and eosin stain; original magnification ×10 (A) and ×400 (B-D).

Because the oncocytic morphology was incompletely developed and the principal differential diagnosis remained between a morphologically atypical IOPN-spectrum lesion and an IPMN with focal oncocytic change, ancillary molecular testing was performed on the resection specimen. An RNA-based next-generation sequencing assay using anchored multiplex polymerase chain reaction (137-gene panel) was performed on bulk tumor tissue, without separate analysis of the oncocytic and non-oncocytic components. The assay identified an ATP1B1::PRKACB fusion joining exon 1 of ATP1B1 to exon 2 of PRKACB. A separately performed hybrid-capture DNA sequencing panel had a standard limit of detection of 5% variant allele frequency, with more than 90% of the targeted coding regions covered at ≥125× according to the assay specifications. No pathogenic or likely pathogenic alterations were identified in KRAS, GNAS, TP53, SMAD4, RNF43, BRAF, or CTNNB1, and no clinically relevant copy-number alterations were detected. Immunohistochemistry was not pursued because RNA-based fusion testing was considered more directly informative for distinguishing an atypical IOPN-spectrum lesion from an IPMN with focal oncocytic change. Taken together with the focal oncocytic morphology, these molecular findings supported classification as a morphologically atypical intraductal oncocytic neoplasm within the IOPN spectrum (Figure 5).

**Figure 5:**
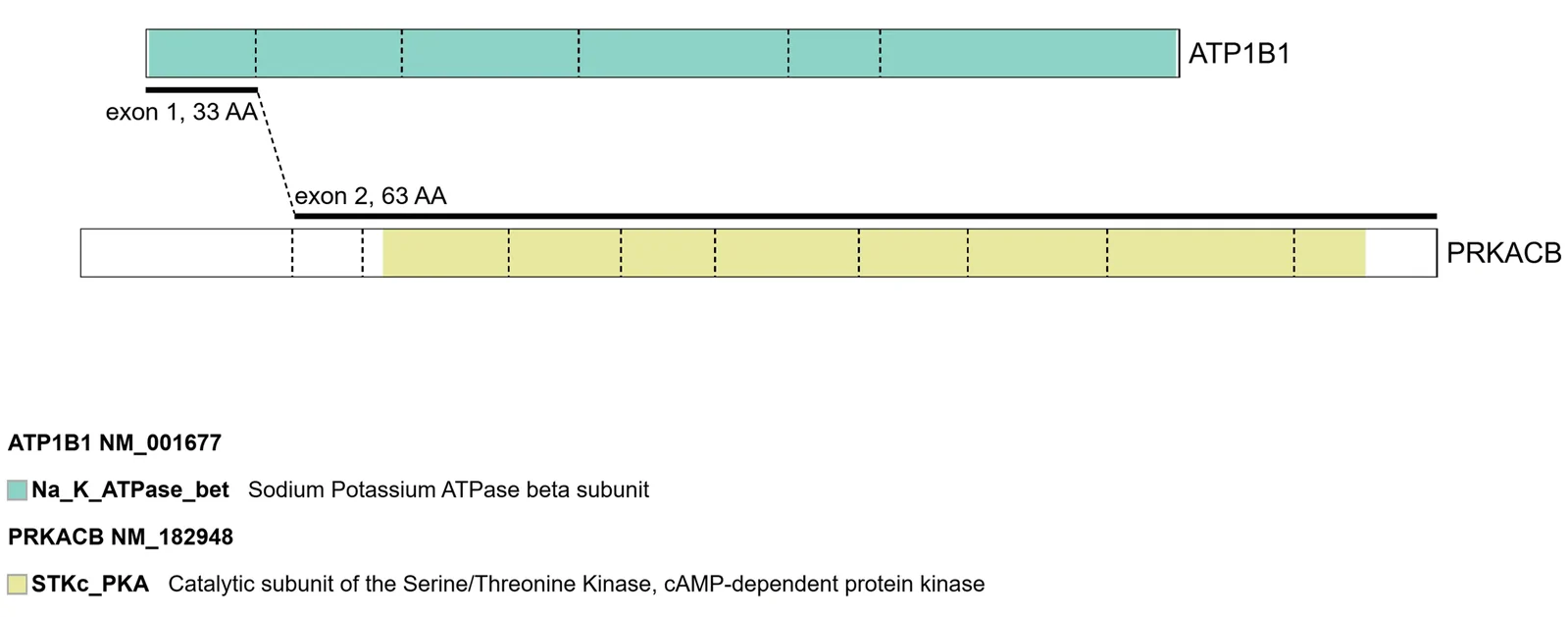
Schematic representation of the ATP1B1::PRKACB fusion. Schematic representation of the ATP1B1::PRKACB fusion. RNA-based next-generation sequencing identified a fusion joining exon 1 of ATP1B1 (NM_001677) to exon 2 of PRKACB (NM_182948). The oblique dashed line denotes the fusion junction observed at the RNA level, vertical dashed lines indicate exon boundaries, and the associated amino acid positions are shown. The fusion retains the cAMP-dependent serine/threonine kinase catalytic domain of PRKACB (yellow), which is predicted to be transcriptionally regulated by the rearranged ATP1B1 promoter [8]. The illustration was generated using ProteinPaint (St. Jude Children's Research Hospital, Memphis, Tennessee) [9]. AA: amino acids.

The postoperative course was complicated by a combined bile and pancreatic leak with an intra-abdominal fluid collection requiring percutaneous drainage. The collection subsequently became infected with *Klebsiella pneumoniae* and was treated with antibiotics. Approximately three weeks postoperatively, the patient had a self-limited episode of bloody drain output. CT angiography showed no active extravasation, and the episode was managed conservatively with observation. At the most recent follow-up, about seven weeks after surgery, pancreatic and biliary drain output had decreased sufficiently to allow drain removal. The postoperative course was also notable for constipation, attributed in part to pancreatic enzyme replacement therapy, which resolved after discontinuation of the medication and standard bowel-regimen management. No additional intervention was required. At this early postoperative follow-up, there was no clinical or radiologic evidence of disease recurrence.

## Discussion

Imaging-based risk stratification and longitudinal stability

IOPN cannot be reliably distinguished from IPMN by imaging alone. Because IOPN is rare, relatively few studies have characterized its radiologic features, and the findings reported to date are not specific to this entity [10,11]. Across published series, IOPNs are often relatively large at diagnosis, with a typical size of approximately 4.5 cm [3,4]. In the 16-case series by D'Onofrio et al., 12 tumors (75%) involved the main duct or showed mixed duct involvement; all four branch-duct lesions measured greater than 3 cm; 10 cases showed main pancreatic duct dilation of at least 10 mm; and 10 contained solid enhancing nodules [11]. Despite the frequent presence of these concerning imaging findings, no statistically significant association was demonstrated between the radiologic predictors evaluated and histologic grade or invasion [11].

The prolonged radiographic stability of the present lesion is also noteworthy. Iranpour et al. reported that resected IOPNs were predominantly complex cystic lesions, frequently associated with main pancreatic duct involvement, cyst size ≥3 cm, and a thin or imperceptible outer wall [10]. Enhancing mural nodules were present in 93% of cases in that series. Serial preoperative imaging obtained at least five months apart was available for only four patients: two lesions remained stable over intervals of five months and one year, whereas two enlarged by 15-17 mm over three to four years; neither of the enlarging lesions contained invasive carcinoma at resection [10]. The authors identified the paucity of serial imaging as a limitation in assessing the temporal evolution of IOPN [10]. In contrast, the present case provides five years of annual surveillance imaging showing no appreciable interval change and no development of a solid component or an enhancing mural nodule. This longitudinal observation adds to the limited available information on the preoperative natural history of IOPN-spectrum lesions.

Morphologic spectrum: conventional and atypical IOPN

Although conventional IOPN has a distinctive histologic appearance, recent studies have broadened the recognized morphologic spectrum of these neoplasms. Tanaka et al. described atypical IOPNs in which the oncocytic component lacked the characteristic complex arborizing architecture or prominent nucleoli, as well as lesions with admixed gastric-, intestinal-, or pancreatobiliary-type IPMN-like epithelium [5]. Itoh et al. similarly demonstrated that oncocytic differentiation may be focal or incompletely developed and that morphologically atypical lesions exhibit greater molecular heterogeneity than conventional IOPN [7]. The present case closely resembles this atypical spectrum: complex arborizing papillae were absent, oncocytic differentiation was focal, and much of the lesion was composed of foveolar-type and pancreatobiliary-type epithelium. Table 1 summarizes the features of conventional IOPN, atypical IOPN-spectrum lesions, and the present case.

**Table 1:** Comparison of conventional intraductal oncocytic papillary neoplasm (IOPN), atypical IOPN-spectrum lesions, and the present case. IPMN: intraductal papillary mucinous neoplasm; IOPN: intraductal oncocytic papillary neoplasm; RT-PCR: reverse transcription-polymerase chain reaction.

Feature	Conventional IOPN	Atypical IOPN-spectrum lesions	Present case
Architecture	Complex arborizing papillae with delicate fibrovascular cores; cribriform and/or solid areas may occur [1,3,4]	Classic arborizing architecture reduced or absent; flat, simple papillary, or IPMN-like areas may be present [5]	Cystic lesion lacking complex arborizing papillae and other fully developed architectural features of conventional IOPN
Cytoplasm	Diffuse, abundant granular eosinophilic (oncocytic) cytoplasm [5]	Oncocytic/eosinophilic differentiation may be attenuated, focal, or incompletely developed [7]	Focal oncocytic differentiation with abundant granular eosinophilic cytoplasm; admixed foveolar-type epithelium with abundant pale mucinous cytoplasm and pancreatobiliary-type epithelium
Nuclei	Large nuclei with a single conspicuous, often eccentric nucleolus [7]	Less conspicuous nucleoli; IPMN-like areas may show tall columnar cells with basally oriented nuclei [5]	Prominent nucleoli within the oncocytic focus; pancreatobiliary-type epithelium showing nuclear stratification, loss of polarity, and marked cytologic atypia
Intraepithelial lumina	Characteristically present [1,3,4]	Not specifically characterized in the cited atypical series	Not described
Companion components	Not typically present	Admixed gastric-, intestinal-, or pancreatobiliary-type IPMN-like components [5,7]	Foveolar-type and pancreatobiliary-type epithelium
Fusion rate	3/3 (100%) by FISH [5]; 32/32 (100%) by RT-PCR [7]	15/19 (79%) by FISH [5]; 13/28 (46%) by RT-PCR [7]	ATP1B1::PRKACB (exon 1 of ATP1B1 joined to exon 2 of PRKACB), detected by RNA-based NGS fusion assay
IPMN-associated genetic and immunophenotypic abnormalities	KRAS mutation 8/32 (25%); GNAS mutation 2/32 (6%); aberrant STK11 expression 1/32 (3%); aberrant p53 expression 0/32 (0%) [7]	KRAS mutation 15/28 (54%, P = .100); GNAS mutation 6/28 (21%, P = .390); aberrant STK11 expression 11/28 (39%, P < .001); aberrant p53 expression 7/28 (25%, P < .001) [7]	KRAS and GNAS: no alterations detected; STK11 and p53 not assessed

Molecular findings as the diagnostic anchor

ATP1B1::PRKACB is one of the recurrent fusions characteristic of IOPN, along with DNAJB1::PRKACA and ATP1B1::PRKACA [6]. In the series by Singhi et al., ATP1B1::PRKACB was identified in 10 of 20 pancreatic IOPNs, while the remaining tumors harbored DNAJB1::PRKACA or ATP1B1::PRKACA fusions [6]. In the systematic review by Paolino et al., a fusion involving PRKACA or PRKACB was identified in all 68 informative cases, with the ATP1B1::PRKACB fusion the most frequent, present in 27 cases (39.7%) [4]. These findings establish recurrent PRKACA/PRKACB rearrangements as a highly characteristic molecular feature of IOPN.

Fusion testing has both strengths and limitations as a diagnostic tool. None of the 126 non-IOPN pancreatobiliary lesions examined by Singhi et al. harbored PRKACA or PRKACB rearrangements, supporting high specificity for IOPN in that cohort [6]. However, the absence of a detectable fusion does not exclude an IOPN-spectrum lesion. PRKACA/PRKACB fusions were not detected in 21% of atypical cases studied by Tanaka et al. and in 54% of atypical oncocytic neoplasms studied by Itoh et al. [5,7]. Atypical lesions may also harbor molecular alterations more commonly associated with IPMN. Itoh et al. found KRAS and GNAS alterations more frequent in atypical than in conventional IOPNs. However, these differences were not statistically significant, whereas aberrant STK11 and p53 immunohistochemical expression was significantly more frequent in atypical lesions [7]. These findings support the concept that morphologically atypical oncocytic neoplasms comprise a molecularly heterogeneous group with overlapping features of conventional IOPN and IPMN [7].

The present case illustrates both the value and the limitations of this molecular approach. Morphology alone did not permit confident classification because oncocytic differentiation was focal and the lesion lacked the fully developed architectural features of conventional IOPN. Detection of the ATP1B1::PRKACB fusion, together with the absence of detectable KRAS and GNAS alterations, strongly supported classification within the IOPN spectrum. However, molecular testing was performed on tumor tissue without separate analysis of the oncocytic and non-oncocytic components. Itoh et al. demonstrated that, in some morphologically mixed lesions, PRKACA/PRKACB fusions may be restricted to the oncocytic component [7]. Therefore, the present analysis cannot determine whether the ATP1B1::PRKACB fusion was present throughout the lesion or confined to the focal oncocytic component. We accordingly favor classification as a morphologically atypical intraductal oncocytic neoplasm within the IOPN spectrum rather than interpreting the fusion alone as evidence of conventional IOPN throughout the lesion.

Preoperative diagnostic pitfalls

Distinguishing IOPN from other pancreatic neoplasms preoperatively can be challenging. In Wang et al.'s 24-case series, the working preoperative diagnosis was pancreatic ductal adenocarcinoma in five of 12 patients (42%) for whom this information was available [3]. Cytomorphologic assessment may therefore play an important role, particularly when imaging lacks distinctive features or abundant mucin is absent. Features supporting IOPN on cytologic specimens include oncocytic cytology, marked cellular complexity, papillary architecture distinct from that of conventional IPMN, and characteristic punched-out intercellular spaces [12].

The anatomic relationship between the lesion and the pancreatic ductal system may also provide useful clues. In a series of three IOPNs evaluated by MRI, Xiang et al. found that each tumor arose within or maintained communication with either the main pancreatic duct or a branch duct. Branch-duct lesions appeared as cystic-solid masses that maintained close continuity with the involved duct, which remained patent at its junction with the main pancreatic duct, rather than simply displacing or compressing the ductal system. Their review also noted that the increased mitochondrial content characteristic of oncocytic cells may contribute to marked 18F-fluorodeoxyglucose avidity on positron emission tomography, potentially creating a false impression of aggressive malignancy [13].

The present case shared some of these anatomic features but differed substantially in its radiographic appearance. Imaging demonstrated a cystic lesion closely related to the pancreatic ductal system, and neither the main pancreatic duct nor the common bile duct showed focal obstruction. Gross examination subsequently confirmed that the lesion arose from and branched off the main pancreatic duct. However, no solid component or enhancing mural nodule developed during five years of surveillance. This contrasts with the cystic-solid branch-duct lesions described by Xiang et al. and with the high frequencies of enhancing mural nodules reported by Iranpour et al. and D'Onofrio et al. [10,11,13]. Because published imaging cohorts have largely consisted of surgically resected IOPNs, lesions with solid or nodular components may be preferentially represented. The present case therefore demonstrates that an IOPN-spectrum lesion may remain radiographically stable and lack the more conspicuous imaging features described in many resected tumors.

Differential diagnosis

The differential diagnosis of a pancreatic intraductal neoplasm with oncocytic or eosinophilic cytoplasm includes pancreatobiliary-type IPMN, acinar cell carcinoma, ITPN, and neuroendocrine tumor [1,12]. Acinar cell carcinoma may mimic IOPN because of its relatively monotonous nuclei, a single prominent nucleolus, and abundant granular cytoplasm; however, acinar neoplasms are generally more mitotically active and more frequently necrotic, do not characteristically form the complex papillary structures of IOPN, and lack the intracellular and extracellular mucin and characteristic cytoplasmic vacuoles seen in IOPN. They also express acinar markers such as trypsin, chymotrypsin, lipase, and BCL10 [12]. Pancreatic neuroendocrine tumors may likewise show oncocytic cytology, with plasmacytoid cells containing granular cytoplasm and eccentric, round nuclei, but typically express neuroendocrine markers such as synaptophysin and chromogranin [12].

ITPN is characterized by predominantly tubular growth, minimal mucin production, uniformly high-grade dysplasia, and usually absent MUC5AC expression [1]. IOPN and ITPN are also molecularly distinct: IOPN is characterized by recurrent PRKACA or PRKACB fusions, whereas ITPN lacks these characteristic rearrangements and more often harbors alterations involving the PI3K pathway, including PIK3CA and PTEN [1]. The distinction between ITPN and intraductal acinar cell carcinoma may be particularly difficult when enzymatic concretions or cytoplasmic zymogen granules are absent. In such cases, immunohistochemical evaluation for acinar differentiation, including trypsin and BCL10, may be helpful [1].

Pancreatobiliary-type IPMN is the principal morphologic differential diagnosis for IOPN because both may show high-grade cytologic atypia and abundant eosinophilic cytoplasm. Features favoring IOPN include relative cytologic monotony, orderly architecture, complex arborizing papillae, and characteristic intraepithelial lumina [1,4]. Paolino et al. emphasized features that help distinguish IOPN from pancreatobiliary-type IPMN with high-grade dysplasia, including relative cellular monotony with little pleomorphism, an orderly epithelial appearance, absence of irregular epithelial tufting, absence of intraepithelial tumor-infiltrating neutrophils, absence of necrobiotic change, and presence of characteristic intraepithelial lumina [4]. Immunophenotypically, MUC6, HepPar-1, and CD117 are more commonly or more strongly expressed in IOPN than in morphologically overlapping pancreatic intraductal neoplasms; IOPN may also express MUC1, whereas MUC2 expression, when present, is generally confined to goblet cells [4,14]. MUC5AC is expressed in the great majority of IOPNs, whereas CDX2 expression is generally absent or restricted to focal goblet-cell differentiation [4,14]. SMAD4 alterations are uncommon in IOPN [4].

In the present case, the morphologic differential favored IPMN. A focal area of oncocytic differentiation, characterized by abundant granular eosinophilic cytoplasm and prominent nucleoli, raised the possibility of IOPN; however, the lesion lacked the complex arborizing papillae and other fully developed architectural features of conventional IOPN. The remainder of the lesion was composed predominantly of foveolar-type and pancreatobiliary-type epithelium, leading to an initial interpretation of IPMN with focal oncocytic change. No immunohistochemical studies were performed in this case. Given the overlapping immunophenotypic features of these related pancreatic intraductal epithelial neoplasms, molecular testing was more informative for the principal classification question. Detection of the ATP1B1::PRKACB fusion, together with the absence of detectable KRAS and GNAS alterations, provided the strongest ancillary support for classification within the IOPN spectrum.

Margin assessment and pseudoinvasion

Margin assessment is an important component of pancreatic intraductal neoplasm evaluation. Xue and Basturk noted that when frozen-section examination of the pancreatic margin shows unremarkable ductal epithelium or low-grade dysplasia, including low-grade IPMN or pancreatic intraepithelial neoplasia, additional resection is generally not indicated if no other lesion is present in the remaining pancreas [14]. In contrast, high-grade dysplasia or invasive carcinoma at the margin warrants consideration of further resection when feasible [14]. In the present case, frozen sections of the pancreatic neck and bile duct margins were negative for high-grade dysplasia or malignancy, and permanent sections showed only incidental low-grade mucinous epithelium at the pancreatic neck margin.

IOPN may also pose substantial difficulty in assessing invasion because of its tendency toward extensive intraepithelial or pagetoid spread into adjacent ducts [3,4]. In the series by Wang et al., 11 of 24 tumors showed morphologic features mimicking invasive carcinoma. These deceptive patterns included extension along adjacent benign ducts dispersed among atrophic lobules, myxoid or edematous stroma surrounding markedly dilated involved ducts that could simulate desmoplasia, and tangential sectioning. Features favoring pseudoinvasion included an overall lobulated architecture, similar stroma surrounding adjacent ducts, and preservation of luminal connections on deeper sections. Unequivocal invasive carcinoma was identified in seven of 24 cases (29%) and was usually present only as microscopic foci [3]. Paolino et al. similarly emphasized that extensive intraductal spread into atrophic lobules can create highly deceptive pseudoinfiltrative patterns [4]. Bakkaloglu et al. noted that this invasion-versus-pseudoinvasion distinction is particularly challenging in IOPN and ITPN relative to IPMN [1]. When invasion cannot be confidently determined, the Verona consensus considers a diagnosis of “indeterminate for invasion” acceptable [15]. Careful assessment of ductal architecture, lobulated contours, stromal characteristics, and preserved luminal connections can help distinguish intraductal spread from true invasion [1,3,15].

Prognosis

Non-invasive IOPN generally has an excellent prognosis after complete surgical resection. In the systematic review by Paolino et al., more than 90% of patients were alive after resection, although follow-up duration was not consistently reported [4]. Wang et al. observed no disease-related deaths among 18 patients with available follow-up; 10-year overall survival was 94%. Overall survival did not differ significantly by invasion status (P = .38), and five-year recurrence-free survival was 93% for non-invasive tumors compared with 66% for invasive tumors. However, this difference did not reach statistical significance (P = .066) [3].

The prognosis of invasive IOPN is less well defined. In a multicenter study of 415 patients with pancreatic intraductal neoplasms, Lucocq et al. identified 20 patients with invasive IOPN; 45% developed recurrence and 60% died during follow-up, with a median overall survival of 24.4 months [16]. Recurrence and overall survival were similar to those of ductal-type IPMN-derived adenocarcinoma, whereas overall survival was significantly worse than that of colloid-type carcinoma [16]. In contrast, Habib et al., in an international multicenter cohort of 879 patients, reported recurrence rates of 25% for IOPN-derived carcinoma and 24% for colloid IPMN-derived carcinoma, compared with 42% for tubular IPMN-derived carcinoma (P < .001) [17]. IOPN-derived carcinoma was also associated with a 74% lower five-year mortality risk than tubular IPMN-derived carcinoma [17]. These findings underscore the heterogeneity of reported outcomes for invasive IOPN and the limitations of drawing firm prognostic conclusions from this rare tumor type.

Management and postoperative surveillance

No IOPN-specific threshold for surgical resection or a dedicated postoperative surveillance protocol has been established, and management is generally extrapolated from guidelines for IPMN and other pancreatic cystic lesions [18-20]. Histologic subtype plays a limited role in preoperative management because the specific epithelial subtype is often unknown before resection, and its independent prognostic significance remains incompletely defined [19]. Consequently, clinicians generally base decisions on cyst morphology, ductal involvement, size, mural nodules, symptoms, and other established risk features rather than on suspected IOPN histology alone [18,19].

Nevertheless, long-term postoperative surveillance should be considered because recurrence can occur after resection. Wang et al.'s systematic review concluded that postoperative management and surveillance of IOPN should be distinguished from those for other pancreatic cystic lesions, while also emphasizing its generally favorable prognosis [20]. More recently, Habib et al. demonstrated that IOPN-derived carcinoma had a longer time to recurrence than colloid and tubular IPMN-derived carcinomas and suggested that this more indolent pattern may support prolonged surveillance [17]. Although the optimal duration and interval of follow-up remain undefined, these observations support continued long-term clinical and radiologic monitoring after resection.

No invasive carcinoma was identified in the present case, placing the patient in the more favorable prognostic group. However, postoperative follow-up remains short and is insufficient to assess long-term oncologic outcome. Given the potential for delayed recurrence in IOPN, continued long-term surveillance is appropriate [17,20].

## Conclusions

This case demonstrates that pancreatic intraductal oncocytic neoplasms within the IOPN spectrum may lack the fully developed architecture and diffuse oncocytic cytology traditionally associated with conventional IOPN. In the present lesion, predominantly foveolar-type and pancreatobiliary-type epithelium with only focal oncocytic differentiation initially favored an indeterminate cystic neoplasm within the IPMN spectrum. Detection of an ATP1B1::PRKACB fusion, together with the absence of detectable KRAS and GNAS alterations, provided important molecular support for classification within the IOPN spectrum. Because molecular testing was not performed separately on the individual morphologic components, the distribution of the fusion within the lesion cannot be established, representing an important limitation. This case also provides uncommon longitudinal documentation of an IOPN-spectrum lesion that remained radiographically stable for five years. When oncocytic differentiation is present but conventional IOPN morphology is incompletely developed, integrated morphologic and molecular assessment can help resolve otherwise difficult classification.
